# The Effect of Non-Thermal Plasma on the Structural and Functional Characteristics of Human Spermatozoa

**DOI:** 10.3390/ijms22094979

**Published:** 2021-05-07

**Authors:** Eva Tvrdá, Daniel Lovíšek, Stanislav Kyzek, Dušan Kováčik, Eliška Gálová

**Affiliations:** 1 Department of Animal Physiology, Faculty of Biotechnology and Food Sciences, Slovak University of Agriculture, Tr. A. Hlinku 2, 949 76 Nitra, Slovakia; evina.tvrda@gmail.com; 2 Department of Genetics, Faculty of Natural Sciences, Comenius University, Ilkovičova 6, Mlynská dolina, 842 15 Bratislava, Slovakia; lovisek.daniel@gmail.com (D.L.); kyzek2@uniba.sk (S.K.); 3 Department of Experimental Physics, Faculty of Mathematics, Physics and Informatics, Comenius University, Mlynská dolina, 842 48 Bratislava, Slovakia; kovacik@fmph.uniba.sk

**Keywords:** non-thermal plasma, spermatozoa, reactive oxygen species, structural integrity, functional activity

## Abstract

Significant antibacterial properties of non-thermal plasma (NTP) have converted this technology into a promising alternative to the widespread use of antibiotics in assisted reproduction. As substantial data available on the specific in vitro effects of NTP on male reproductive cells are currently missing, this study was designed to investigate selected quality parameters of human spermatozoa (n = 51) exposed to diffuse coplanar surface barrier discharge NTP for 0 s, 15 s, 30 s, 60 s and 90 s. Sperm motility characteristics, membrane integrity, mitochondrial activity, production of reactive oxygen species (ROS), DNA fragmentation and lipid peroxidation (LPO) were investigated immediately following exposure to NTP and 2 h post-NTP treatment. Exposure to NTP with a power input of 40 W for 15 s or 30 s was found to have no negative effects on the sperm structure or function. However, a prolonged NTP treatment impaired all the sperm quality markers in a time- and dose-dependent manner. The most likely mechanism of action of high NTP doses may be connected to ROS overproduction, leading to plasma membrane destabilization, LPO, mitochondrial failure and a subsequent loss of motility as well as DNA integrity. As such, our findings indicate that appropriate plasma exposure conditions need to be carefully selected in order to preserve the sperm vitality, should NTP be used in the practical management of bacteriospermia in the future.

## 1. Introduction

Due to a variety of environmental, occupational and lifestyle factors, the number of couples that benefit from assisted reproduction (AR) is increasing. High-quality spermatozoa and oocytes equally contribute to the success of AR, which is why it is essential to collect and process structurally and functionally competent gametes which will subsequently impact the embryonic development and viability [1,2]. In particular, there is a need to constantly improve protocols for semen processing in order to prevent unnecessary sperm damage before cryopreservation, artificial insemination or in vitro fertilization.

As pointed out by a variety of studies, bacterial contamination of ejaculates has become an essential contributor to excessive sperm degradation in AR clinics. Bacterial adherence to the surface of male gametes followed by sperm agglutination [3,4], motility loss [3,5,6] and an impaired acrosome reaction [5,6] have been observed in bacteria-infested sperm cultures. Furthermore, it has been reported that the use of contaminated sperm suspensions may lead to oocyte degeneration and transmission of infections to the recipients [7].

The advantages of supplementing antibiotics to semen extenders as well as preparation, cryopreservation and IVF media have been questioned for a couple of years now. Despite the ability of antibiotics to prevent bacteriospermia, an increased number of reports have emerged emphasizing on the potentially toxic effects of traditional antibiotics on the sperm and embryo vitality [8,9,10]. These findings, coupled with a dramatically increasing bacterial resistance to antibiotics, have contributed to the need for finding appropriate alternatives to reduce their widespread use during semen handling.

The circumstances mentioned above have led us to the idea that non-thermal plasma (NTP), with the ability to achieve enhanced gas-phase chemistry and high efficiency of microbial inactivation without increasing the gas temperature, could theoretically be used as a replacement to antibiotics for the treatment of semen. The antimicrobial effects of NTP are among the best investigated [11]. Advancements in the design of NTP systems have also underlined their potential for several applications in medicine and biology, such as wound healing, promotion of cell proliferation, tissue regeneration and cancer treatment [12,13,14]. Furthermore, preliminary studies suggest that NTP could be used to stimulate the motility of spermatozoa collected from asthenozoospermic patients [15,16] and avian species [17,18].

The use of diffuse coplanar surface barrier discharge (DCSBD) is also a perspective NTP technology, which has been successfully applied for the management of bacterial decontamination of biological materials [19]. The portable NTP generator used in our experiments—RPS40—is based on DCSBD technology. The plasma generated by the RPS40 source exhibits its antibacterial effects most likely due to the production of reactive oxygen species (ROS). However, an equally important aspect of its practical application in biological research lies in the understanding of its impact on human cells which may be in close contact with NTP during treatment. Moreover, spermatozoa appear to be a suitable model for monitoring the NTP effects due to their specifically condensed DNA as well as a lack of repair mechanisms and antioxidant protection. These unique physiological characteristics play a role in an increased sperm susceptibility to oxidative stress, whose mechanisms of action on the male gamete are still not fully understood [20].

As such, this study aimed to determine the potential impact of DCSBD-type NTP on human spermatozoa in vitro.

## 2. Results

The effects of NTP on the proportion of motile spermatozoa are detailed in Table 1 and Table 2. Immediately following treatment, significantly lower motility (MOT) and progressive motility (PROG) were detected in the experimental groups subjected to a 60 s (*p* < 0.05 in the case of MOT; *p* < 0.01 concerning PROG) and a 90 s (*p* < 0.01 for MOT; *p* < 0.001 concerning PROG) exposure to NTP. Possible detrimental effects of an extended NTP treatment on the motion characteristics were recorded after 2 h post-exposure. A significantly decreased MOT (*p* < 0.05) as well as PROG (*p* < 0.001) were detected in the experimental group exposed to 60 s of NTP in comparison with the control. Furthermore, an abrupt decline in the motility characteristics was recorded in the group previously subjected to 90 s of NTP when compared to the control group (*p* < 0.001). No significant changes to MOT or PROG were detected in the samples exposed to 15 s or 30 s of NTP (Table 1 and Table 2).

Negative effect of NTP doses of 60 s and 90 s on the secondary motility parameters serving as predictors for the overall fertility and chances of pregnancy, represented by the path velocity (VAP) and curvilinear velocity (VCL) revealed itself shortly following treatment in the form of a significant decline of both markers (*p* < 0.001) in comparison with the control group. Lower exposure times to NTP (15 s and 30 s) had no significant effects neither on VAP (Table 3) nor on VCL (Table 4).

Assessment of both characteristics 2 h post-NTP treatment showed further decline of VAP as well as VCL which remained significant in the samples subjected to 60 s as well as 90 s (*p* < 0.001) of NTP when compared with the control.

The analysis of motion characteristics necessary for cervical mucus penetration, including the straight line velocity (VSL), straightness (STR) and lateral amplitude (ALH) reveal that amongst the observed parameters, VSL was the most affected by high NTP exposition times. A significant decrease of VSL was observed immediately (0 h) following exposure do 60 s and 90 s of NTP (*p* < 0.001) in comparison to the control group. A similar observation was recorded 2 h following NTP treatment (Table 5).

In the meantime, immediate NTP treatment had no significant impact on STR (Table 6) or ALH (Table 7); nevertheless, at 2 h, a significant decline of both parameters was observed in the experimental groups previously subjected to 60 s (*p* < 0.05) as well as 90 s of NTP when compared to the control group.

Finally, assessment of the beat cross frequency (BCF) and linearity (LIN) as parameters estimating the ability of spermatozoa to move throughout the uterus and Fallopian tubes indicates that among these, BCF was more affected by the NTP treatment than LIN. A significant decrease of BCF was recorded in the experimental groups subjected to NTP for 60 s as well as 90 s (*p* < 0.05; Table 8) when compared to the control group immediately following NTP treatment as well as 2 h post-exposure.

In case of LIN, at 0 h (Table 9), a decrease of the parameter was observed, particularly in case of NTP doses of 60 s and 90 s, although all changes were insignificant. A similar observation was recorded 2 h following NTP treatment. A decline of LIN was recorded in case of the experimental groups exposed to 60 s of NTP and this trend became more profound following the longest plasma exposure time (90 s). Nevertheless, no significant changes were detected.

Qualitative changes in the sperm membranes related to the process of apoptosis or necrosis were determined using the Annexin V (AV) and propidium iodide (PI) fluorescent staining protocol 0 h and 2 h after 0 s, 15 s, 30 s, 60 s and 90 s of NTP treatment. It may be observed that immediately following exposure to NTP, the proportion of apoptotic (Table 10) and necrotic spermatozoa (Table 11) increased in a dose-dependent manner when compared to the control. The most significant apoptotic or necrotic disruption of the sperm membranes at 0 h was observed in case of the experimental group subjected to a 90 s NTP treatment (*p* < 0.05 in case of AV-positivity; *p* < 0.01 with respect of PI-positivity).

An interesting observation was noted in the case of the changing ratio of early apoptotic cells to necrotic cells. In the unaffected sample as well as following NTP treatment of 15 s and 30 s, a slight predominance of cells in early apoptosis was noted (Table 10). However, this trend reversed in favour of necrotic (or late apoptotic) cells at a 60 s dose and was even more pronounced at 90 s (Table 11).

The analysis at 2 h following NTP treatment revealed more radical membrane damage in comparison to the assessment immediately following exposure to NTP. A significantly higher percentage of cells exhibiting signs of early apoptosis (Table 10) as well as late apoptosis or necrosis (Table 11) was detected in the groups previously exposed to 60 s (*p* < 0.05) and 90 s (*p* < 0.001 for AV-positivity; *p* < 0.01 in case of PI-positivity).

At 0 h (Table 12), a slight increase in the mitochondrial activity of the experimental group subjected to the lowest NTP exposure time (15 s) was observed. By increasing the plasma exposure time to 30 s and 60 s, the mitochondrial function recorded was almost equal to the control values. At the maximum exposure time (90 s), the sperm mitochondrial activity slightly decreased when compared to the control. All changes in the sperm metabolic function were insignificant.

A similar observation was recorded 2 h following NTP treatment (Table 12). A decrease of the mitochondrial metabolism was noted in case of the experimental group exposed to 60 s of NTP and this decline became even more pronounced following the highest plasma exposure time (90 s). Nevertheless, no significant changes were recorded.

Inversely, the JC-1 assay revealed the first differences in the mitochondrial membrane potential (Δψm) immediately following a 90 s exposure to NTP, reflected in a significantly lower mitochondrial vitality (*p* < 0.05) when compared to the control (Table 13). Disrupting effects of NTP on Δψm were observed following 2 h as well. At the end of the experiment, the lowest Δψm was detected in the experimental groups subjected to a 60 s (*p* < 0.05) and a 90 s exposure (*p* < 0.01) to NTP (Table 13).

The effect of NTP on the sperm nuclear DNA manifested itself immediately following treatment in the form of an increased rate of DNA fragmentation. Lower exposure times to NTP (15 s and 30 s) had no significant effects on the rate of DNA damage, while a significant increase of cells exhibiting a fragmented DNA molecule was observed at doses of 60 s and 90 s (*p* < 0.001) when compared to the control. Interestingly, by increasing the NTP dose from 60 s to 90 s, the DNA fragmentation index did not increase further (Table 14).

Assessment of the DNA fragmentation index after 2 h post-NTP treatment led to slightly different results in comparison to 0 h. Substantial changes occurred at a dose of 60 s and 90 s caused significant DNA damage to the male gametes, which was as twice as high in comparison to the unaffected control (*p* < 0.001). Notable dose-dependent differences in the progress of sperm DNA damage were also recorded concerning NTP exposure times of 60 s and 90 s, which were not observed at 0 h.

At 0 h, ROS production in the experimental groups increased proportionally depending on the increasing dose of NTP (Table 15). A slightly increased ROS concentration was observed following a 15 s and 30 s exposure to NTP when compared to the untreated sample. Nevertheless, a significant rise in the ROS production was recorded in the experimental groups subjected to NTP for 60 s (*p* < 0.05) and 90 s (*p* < 0.01) in comparison to the control group. ROS-promoting effects of NTP were observed following 2 h post-treatment, as significantly higher ROS amounts were found in the experimental groups subjected to NTP for 60 s (*p* < 0.05) as well as 90 s (*p* < 0.01) against the control.

Immediately following treatment (0 h), a slightly increased superoxide concentration was observed in all experimental group subjected to NTP (Table 16).

At 2 h following exposure to NTP, an increase of superoxide generation was noted particularly in the case of the experimental group subjected to 60 s of NTP and this rising tendency became even more distinct following the highest exposure time to NTP (90 s). Nevertheless, no significant differences against the control were recorded.

The thiobarbituric acid reactive substances (TBARS) assay revealed that NTP exhibits the ability to induce ROS-inflicted damage to the sperm lipids (Table 17). A significantly increased malondialdehyde (MDA) concentration was detected immediately (0 h) following a 90 s exposure to NTP (*p* < 0.05) in comparison with the untreated control. After 2 h, a significantly higher LPO was recorded in the case of samples subjected to a 60 s (*p* < 0.05) and 90 s (*p* < 0.01) NTP treatment. No significant changes in the samples exposed to NTP for 15 s and 30 s were observed.

## 3. Discussion

When examining the potential application of NTP in biology and medicine, particular attention must be devoted to understanding how specifically NTP affects the cell and which of its components could be responsible for any observed changes in the cell behaviour. Describing this interaction at the molecular level may be complicated due to the intricate nature of NTP and the heterogeneity of biological targets. At the same time, we must take into account that all components of the plasma travel a certain distance and often trespass different environments (e.g., air/water interface) when affecting a given subject, which creates space for various interactions that can lead to the formation of new components [21].

As this study focused primarily on the changes of the structural integrity and functional activity of spermatozoa in response to NTP, we focused on the exclusion of any additional factors that could have a potential impact on the obtained data. Previous studies on the effect of plasma on DNA in aqueous solutions partially helped us to select an appropriate culture medium for the collected semen samples. It has been suggested that complex media could interact with the plasma components (particularly ROS) [22] or change their properties (such as pH) in response to NTP treatment [23]. Similarly, data collected from the chemiluminescent assay for ROS quantification should be interpreted with caution due to a variety of factors that may affect the signal obtained, such as the medium composition or its pH [24]. As such, we chose the simplest possible medium in the form of saline to dilute the ejaculates.

A plasma device that actively generates NTP is characterized by an unmistakable blue-violet light (UV) and a strong odour of ozone. It is well known that these two components are biologically active. However, it is debatable to what extent and whether UV and/or ozone from the plasma could contribute to its overall effect. Lunov et al. [25] compared the impact of NTP and ozone itself on selected bacteria (*Pseudomonas aeruginosa* and *Staphylococcus aureus*) as well as mammalian cell lines (3T3 fibroblasts and glioma cells). The authors confirmed the inhibitory effects of NTP as well as ozone on the bacteria. However, it was found that the cytotoxic effect of ozone alone was stronger and faster in comparison to a heterogenic NTP in case of the tested cell lines. The proposed mechanism of ozone toxicity lies in the induction of a rapid accumulation of ROS in the cell, which could lead to cell death, particularly necrosis. In this study, we also observed an immediate inhibitory effect of prolonged exposure to NTP on the selected sperm quality parameters, which could indicate that the plasma generated by the RPS40 device could contain a significant amount of ozone. However, the interpretation of the results in this direction is complicated since the exact composition of the plasma produced at a specific moment is difficult to be determined.

The presence of even relatively low concentrations of ozone may be detected by the sense of smell [26]. Nevertheless, the perception of ozone during the treatment of samples does not necessarily mean that it contributes significantly to the detrimental effects of high NTP doses on male reproductive cells. On the other hand, Gradil et al. [27] demonstrated that even a relatively low dose (5 μg/mL) of ozone exhibited spermicidal effects on bovine specimens. In this sense, we may agree with the authors, that the cause could lie in damage to the cytoplasmic membrane due to oxidative stress, or a decreased availability of ATP or cAMP for the motility process. However, by summarizing the data available so far, we cannot assess the extent to which ozone from the RPS40 plasma generator contributes to our observations. Therefore, we cannot rule out its possible application in andrology.

UV radiation from plasma seems to be relevant mainly because of its inhibitory effects on bacteria [28]. In eukaryotes, however, the contribution of UV to the overall impact of NTP is unknown, some authors even claim that it is negligible [12,29]. In order to deny the influence of UV, it is necessary to clarify if and how it affects sperm survival. UV radiation is defined by the wavelengths of the electromagnetic spectrum in the range from 100–400 nm. Most studies looked at the effects of shorter wavelength UV radiation because it interacts easily with DNA and induces ROS overproduction, thus exhibiting large genotoxicity [30,31].

The effect of UV-C radiation (254 nm) was tested on human spermatozoa. These were resuspended in a nutrient medium and treated at a distance of 4 cm for 0, 10, 20 and 30 min, corresponding to UV-C doses of 0, 7.92, 15.84 and 23.76 kJ/m^2^. Similarly to our results, high doses of UV-C reduced the sperm motility by damaging membranes through LPO [30]. Other reports compared the effect of UV radiation on spermatozoa obtained from fertile and infertile men and looked at whether they differed in their susceptibility to DNA damage by UV radiation. Using a neutral comet assay, they found that after 15 min of UV-C irradiation (254 nm) at a source distance of 1 m, the rate of DNA fragmentation increased slightly more in the specimens of infertile men.

Nevertheless, DNA damage was also reported in samples collected from fertile subjects [31]. Interestingly, UV-A (365 nm) radiation with lower energy, which is not primarily absorbed by the DNA molecule, also exhibited a damaging effect on male gametes. UV-A treatment of human spermatozoa resulted in a gradual loss of their motility and consequently in a reduction of their overall survival. The explanation could lie in the induction of oxidative stress by this type of radiation [32]. Summarizing the studies mentioned above, a relatively wide spectrum of UV radiation exhibits adverse effects on essential sperm parameters such as motility or DNA and membrane integrity. We recorded similar results, particularly concerning high doses of NTP. Nevertheless, a definitive conclusion cannot be postulated, as specific information on the RPS40 electromagnetic spectrum still needs to be investigated. Furthermore, our specimens were subjected to a complete plasma, without any filters that would separate its individual components and thus allow us to assess their separate effects.

In the context of the biological effects of plasma on living cells, NTP can be considered as an inducer of oxidative stress. Not only is plasma itself a source of ROS, but it induces their further production in response by the cells [33]. A similar property is characteristic of hydrogen peroxide (H_2_O_2_), which belongs to the ROS family. Still, in the intracellular environment, it can become a source of other free radicals due to the Fenton and Haber-Weiss reactions. Furthermore, it is a molecule with a high affinity to lipids.

The effect of oxidative stress on human spermatozoa induced by H_2_O_2_ has been tested by several authors [34,35,36]. All studies revealed that exogenously administered H_2_O_2_ reduced the sperm motility as well as mitochondrial activity, increased the total intracellular concentration of ROS and the degree of DNA fragmentation. This intracellular rise in ROS amounts can be explained by direct diffusion of H_2_O_2_ across the membranes or its incorporation into intracellular pro-oxidative processes. ROS overproduction is considered to be a prime cause of a decreased motility. In addition, high ROS concentrations are associated with peroxidation of membrane lipids and a subsequent loss of the selective permeability and stability of membranous structures. A prooxidant intracellular signalling machinery will be initiated, in which highly reactive LPO end-products may directly damage critical biomacromolecules as well as whole organelles, such as mitochondria [37], which may subsequently become a significant source of ROS themselves, intensifying the risk for irreversible oxidative damage to the cell [34,36]. Oxidative stress is also considered to be a major cause for DNA damage in spermatozoa [35].

Looking at the results obtained from our experiments, several trends can be observed, which may provide more clarification to the NTP impact on human spermatozoa. Our first observation is that high NTP doses exhibited a detrimental impact on almost all observed sperm parameters. The only exception was the mitochondrial activity assessed by the MTT test and the intracellular superoxide production. Both seemed to be unaffected by even high NTP exposure times, although a tendency indicating damage to the mitochondrial machinery could be observed following 2 h post-NTP treatment. The dependence of the sperm kinetic system on ATP, which is by and large created by oxidative phosphorylation in mitochondria, has been proven on numerous occasions [38]. In addition, these parameters were correlated in several earlier studies [36,39,40,41]. On the other hand, an interesting explanation for reduced sperm motility could be provided by a mitochondrial-independent mechanism, which consists in direct oxidative damage to the contractile apparatus of the flagellum (specifically damage to the axoneme or flagellar envelope structures) or in a reduced ability of the sperm cell to actually take advantage of ATP. This hypothesis is based on an earlier study commenting that induced oxidative stress did not cause a change in the activity of the respiratory chain or the amount of intracellular ATP [42]. Similar conclusions are summarized in a report on the effects of oxidative stress generated by the xanthine/xanthine oxidase system on stallion spermatozoa [43]. To provide clarification to these contradictory statements, besides the MTT test, we chose to evaluate the mitochondrial function using the JC-1 assay. In this case, we observed a time- and dose-dependent decrease of the mitochondrial function, which mirrored the decline of the motility characteristics obtained from the CASA analysis. As such, we may speculate that the first cellular components affected by high doses of NTP are the membranous structures of the sperm cell. In addition, the plasma membrane, the outer mitochondrial membrane as a representative of the mitochondrial membrane potential may become a suitable target for the reactive components of NTP. Thus, decreased sperm motility observed immediately following NTP treatment may be a consequence of a disrupted intracellular milieu caused by the membrane destabilization as well as other mitochondria-independent mechanisms as indicated earlier. At later stages, potential alterations to the inner mitochondrial membrane may be expected, leading to a decrease of the activity of the electron transport chain and subsequent ROS leakage from damaged mitochondria, contributing to further sperm immobilization. In addition to the membrane disintegration, peroxidation of membrane lipids takes place due to the high content of polyunsaturated fatty acids in the sperm membranes, which are highly susceptible to oxidative damage [37,44]. Lastly, DNA fragmentation could be attributed to a high vulnerability of the sperm genetic material to oxidative stress. Although the DNA molecule is, in theory, well protected by high levels of nuclear condensation [35,45], by trespassing a critical ROS threshold, subsequent oxidative insults are unavoidable and the resulting damage is impossible to be reversed, as the male gamete lacks a cytoplasm with proper antioxidant or repair mechanisms.

Another observation from this study is that the effects of high plasma doses manifested themselves immediately following treatment on all measured parameters, without the need for preincubation. This suggests that some of the physical components of plasma, which may be characterized by such immediate action, could be responsible for the detected changes in the sperm vitality [30]. While UV, as a critical component of NTP, has not gained significant support from the scientific community as discussed earlier [12,29], this acute toxicity could be indeed caused by ozone, which is a ROS. It could be responsible for increased oxidative pressure on living cells [25]. Accordingly, we detected an increased amount of ROS in the specimens exposed to NTP, which could have been responsible for elevated damage to the sperm DNA and lipids, as suggested by a number of earlier studies [46,47,48]. Nevertheless, we must still emphasize on the fact that it is difficult to determine which of the physical or chemical components of the plasma could be the key one in evoking such a biological reaction. Hence, we may speculate about a synergistic effect of the individual components [49].

The last important observation from our study is the fact that NTP had a significant negative effect on the selected sperm parameters at higher doses exclusively, specifically at 60 and 90 s of exposure. No significant positive or negative impact of the plasma was observed in case of a lower exposure time. It is known that the toxic effects of an equal NTP dose are higher in the case of prokaryotic cells when compared to eukaryotic cells [50]. Assuming lower NTP doses used in our experiments are effective enough to exhibit substantial antibacterial effects, the RPS40 plasma generator could become an interesting strategy to at least decrease the bacterial contamination of semen samples. Nevertheless, to reach a definitive conclusion, further studies on the effects of NTP generated by the RPS40 instrument on selected uropathogenic bacteria or contaminated semen samples are crucial.

## 4. Materials and Methods

### 4.1. RPS40 Plasma Generator Characteristics

**Figure 1:**
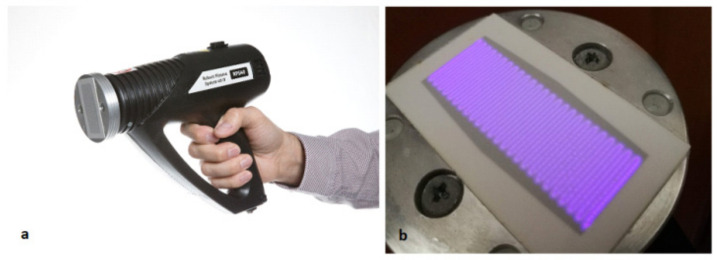
The photos are illustrating RPS40 plasma system, the general view of the handheld device (**a**) and the view of the macroscopically homogeneous DCSBD plasma layer generated in ambient air (**b**).

Non-thermal plasma was generated by the diffuse coplanar surface barrier discharge installed in a small handheld; portable ambient air plasma system made by Roplass s.r.o. (Brno, Czech Republic) under the designation RPS40 (Figure 1a).

This type of plasma system is a minimized version of the classical DCSBD plasma source [51] which has been successfully investigated and used for surface treatment of a wide range of materials [52,53,54,55,56] including the treatment of the plant seeds [19,57,58,59] and application for bio-decontamination [60,61,62] in recent years. The electrodes system of RPS40 consists of an alumina plate with 25 pairs of parallel conductive strips with the width 0.5 mm and separated from each other by 0.5 mm gap and is supplied by a sinusoidal high voltage with a frequency ~28 kHz and power input of 40 W. To prevent the undesirable sparking between the adjacent electrodes, these are isolated from each other by a thin (0.15 mm) dielectric layer. As shown in Figure 1b, RPS40 generates on the surface of the alumina plate with the dielectric layer a diffuse, macroscopically homogeneous plasma layer with the dimensions of 50 mm × 20 mm, whereas the effective thickness in ambient air is 0.2–0.3 mm. During the operation, the electrode system is cooled down by passive heat dissipation through an aluminium housing, which is simultaneously cooled by a fan. Because of the effective cooling of the electrode system, the temperature of the alumina plate was less than 60 °C for the maximum operation time. The implementation of the RPS40 or standard DCSBD plasma source into the clinical environment is undemanding. It does not require compliance with any particular operating conditions. No special gases are required, as the treatment can be carried out in ambient air. DCSBD plasma systems should be mounted in the holder for adjustment the optimised distance during the treatment and placed in a fume hood to exhaust ozone and nitrogen oxides. For powering RPS40 and a standard DCSBD plasma source, an electrical network with a 230 V (AC) voltage is needed.

### 4.2. Sample Collection and Treatment

Semen samples for this study were obtained from sixty healthy donors (age of 24.6 ± 2.1 years). The inclusion criteria for the donors were: (1) normal semen quality parameters accommodating the World Health Organization (WHO) guidelines (volume ≥ 1.5 mL, concentration ≥ 15 million/mL, motility ≥ 40% and morphology ≥ 4%); (2) no sexually transmitted infections; and (3) may or may not have initiated a pregnancy in the past [63]. In order to trespass possible difficulties associated with potential limitations of the chemiluminescent ROS quantification, only ejaculates free from leukocytes were accepted for subsequent experiments. All donors provided informed consent to use their specimens for the purposes of this study. All procedures performed in the study involving human participants were in accordance with the ethical standards of the institutional and/or national research committee and with the 1964 Helsinki Declaration and its later amendments or comparable ethical standards. This article does not contain any experiments with animals performed by any of the authors. The samples were collected following 2–3 days of abstinence and allowed to liquefy for 30 min at 37 °C before further processing. Nine samples were excluded for not accomplishing the WHO criteria.

Following liquefaction, the samples were diluted in physiological saline solution (B. Braun Melsungen AG, Melsungen, Germany) to a final concentration of 10 million/mL, placed into 6-well plates and subjected to NTP treatment using the RPS40 device. The distance of the thin plasma layer from the samples was set to 2 cm, with exposure times of 0 s (control), 15 s, 30 s, 60 s and 90 s. Selected experimental assessments of the sperm quality were performed immediately following the NTP treatment (0 h) and after a 2 h culture at 37 °C.

### 4.3. CASA (Computer-Assisted Sperm Analysis)

The CASA analysis was performed with the HTM TOX IVOS II. system (version 14.0; Hamilton-Thorne Biosciences, Beverly, MA, USA). Ten μL of each sample were transferred to the Makler counting chamber (10 μm depth; Sefi Medical Instruments, Haifa, Israel), which was subsequently placed into the heated (37 °C) microscopic chamber of the CASA instrument. The output data were obtained using the Animal motility program (Hamilton-Thorne Biosciences, Beverly, MA, USA) [64]. In the case of this study, we were particularly interested in the overall motility (MOT; percentage of spermatozoa with a motility ≥ 5 µm/s; %) and progressive motility (PROG; percentage of spermatozoa with a motility ≥ 40 µm/s; %). Secondary motility characteristics, including path velocity (VAP; µm/s), straight line velocity (VSL; µm/s), curvilinear velocity (VCL; µm/s), lateral amplitude (ALH; µm), beat frequency (BCF; Hz), straightness (STR; %) and linearity (LIN; %) were evaluated as well.

### 4.4. Membrane Integrity

The commercially available Annexin-V-FLUOS kit (Roche Applied Science, Basel, Switzerland) was used to evaluate the membrane stability of the control and experimental samples. Using a dual staining with Annexin-V (AV) and propidium iodide (PI), it is possible to distinguish 3 categories of cells: cells in the early stage of apoptosis, which are in principle still alive, however, with an initial loss of selective membrane permeability (AV-positive/PI-negative), dead cells in necrosis or late-stage apoptosis with a perforated membrane (AV-positive/PI-positive) and, finally, living cells with an intact membrane that do not exhibit a signal for AV or PI [65].

For this assay, 10^6^ spermatozoa were transferred into Eppendorf tubes. Subsequently, 50 μL incubation buffer, 1 μL AV and 1 μL PI were added to each sample. The tubes were covered with foil and incubated for 30 min at 37 °C. Following incubation, the samples were stained with 10 μL DAPI (4′,6-diamidino-2-phenylindole; Sigma-Aldrich, St. Louis, MO, USA; 1 μM in PBS), pipetted into a black opaque 96-well plate and the fluorescence intensity for each stain was quantified using a combined spectro-fluoro-luminometer (Glomax Multi^+^; Promega Corporation, Madison, WI, USA). The data are expressed as % apoptotic cells and % necrotic cells, respectively.

### 4.5. Mitochondrial Activity

The MTT test is a colorimetric method designed to determine the viability of eukaryotic cells by measuring their mitochondrial metabolic activity. The principle of the technique is based on the reduction of the yellow MTT tetrazolium salt to blue-violet, water-insoluble formazan crystals by the enzymatic activity of mitochondrial dehydrogenases, primarily by the succinate dehydrogenase complex of the respiratory chain of functional mitochondria within an active cell [66].

First, 200 μL of the control and experimental samples were transferred from the culture plates into a 96-well plate. Subsequently, 20 μL of the MTT tetrazolium (Sigma-Aldrich, St. Louis, MO, USA) previously dissolved in Dulbecco’s PBS (phosphate-buffered saline without Ca^2+^ or Mg^2+^; Sigma-Aldrich, St. Louis, MO, USA) were added to each specimen and the samples were incubated at 37 °C for one hour. The reaction was stopped with 80 μL of acidic isopropanol (Centralchem, Bratislava, Slovakia). The amount of formazan was determined spectrophotometrically (Multiskan FC microplate photometer; Thermo Fisher Scientific Inc., Waltham, MA, USA). The absorbance was measured at 570 nm (maximum for formazan) versus 620 nm (reference values). The values collected from the experimental groups are expressed in percentage of the control, which was set to 100% [41].

### 4.6. Mitochondrial Membrane Potential

In order to have a closer look on the sperm mitochondrial behaviour following exposure to NTP, the mitochondrial membrane potential (ΔΨm) was examined with the Mitochondrial Membrane Potential Assay Kit (Cayman Chemical, Ann Arbor, MI, USA). The protocol takes advantage of the fluorescent JC-1 cationic dye, which binds to the mitochondria and adjusts its fluorescent features depending on its aggregation. In the case of cells with healthy mitochondria with a high ΔΨm, JC-1 complexes emerge with red fluorescence. In the cells with disrupted mitochondria exhibiting a low ΔΨm, the JC-1 dye remains in its monomeric form with green fluorescence.

One million cells were transferred into Eppendorf tubes, the volume of the cell suspension was adjusted to 100 μL with PBS and subsequently stained with 5 μL JC-1 working solution. Following incubation (20 min, 37 °C, dark conditions) the stained suspensions were washed twice with PBS, transferred to a black opaque 96-well plate and the fluorescence intensity for the JC-1 monomers and polymers was quantified using a combined spectro-fluoro-luminometer (Glomax Multi^+^; Promega Corporation, Madison, WI, USA). The resulting ΔΨm is expressed as the ratio of JC-1 complexes to JC-1 monomers (red/green ratio) [67].

### 4.7. Global ROS Production

The chemiluminescent method to determine the amount of ROS in this study is based on a direct quantification of intracellular as well as extracellular ROS due to a non-specific reaction of the probe (luminol) with oxidizing agents (ROS) within the cell or its surroundings [68].

One hundred μL of the control or experimental cell suspension were transferred to a 96-well plate and subsequently treated with 2.5 μL of 5 mM freshly prepared luminol (Sigma-Aldrich, St. Louis, MO, USA). One hundred μL PBS served as the experimental blank, while the negative control consisted of 100 μL PBS and 2.5 μL luminol. The positive control consisted of 100 μL PBS, 12.5 μL H_2_O_2_ (33%; Sigma-Aldrich, St. Louis, MO, USA) and 2.5 μL luminol. The luminescence was measured with a combined spectro-fluoro-luminometer (Glomax Multi^+^; Promega Corporation, Madison, WI, USA). The intensity of the light in the wells was evaluated by counting photons within 15 1-min cycles. The experimental results are expressed in relative light units per second per million sperm (RLU/s/10^6^ sperm) [67].

### 4.8. Superoxide Production

The NBT test is a colorimetric protocol devised to estimate the extent of the superoxide production within the cell. The experimental approach of the technique is based on the intracellular reaction between nitroblue tetrazolium chloride and superoxide, leading to the formation of blue formazan crystals [69].

First, 200 μL of the control and experimental samples were pipetted into a transparent 96-well plate. Subsequently, 100 μL of the NBT tetrazolium (Sigma-Aldrich, St. Louis, MO, USA) previously dissolved in Dulbecco’s PBS containing 1.5% DMSO (Sigma-Aldrich, St. Louis, MO, USA) were added to each cell suspension. The samples were incubated at 37 °C for one hour, subsequently centrifuged (300× *g* for 10 min) and washed twice with PBS. The reaction was stopped with 2 M potassium hydroxide (KOH; Centralchem, Bratislava, Slovakia) dissolved in DMSO. The amount of formazan was quantified with the Multiskan FC microplate photometer (Thermo Fisher Scientific Inc., Waltham, MA, USA). The absorbance was measured at 570 nm versus 620 nm and the data obtained from the experimental groups are expressed in percentage of the control, which was set to 100% [40].

### 4.9. DNA Fragmentation

Sperm DNA fragmentation was assessed with the Halosperm^®®^ commercial kit (Halotech DNA, Madrid, Spain). Twenty μL of each control and experimental semen sample were mixed with low-melting point agarose provided by the kit. The semen-agarose mix was transferred onto a microscopic slide pre-coated with agarose, covered with a coverslip and cooled down to 4 °C to solidify the agarose. The coverslips were then gently removed and the slides immersed into an acid solution for 7 min. Subsequently, the slides were subjected to a lysis solution for 20 min and washed with distilled water for 5 min. Finally, the slides were dehydrated in 70% and 100% ethanol for 2 min each and air-dried.

For the evaluation, the slides were stained with Sybr-Green (2 μg/mL) (Sigma Aldrich, St. Louis, MO, USA)/Vectashield (Vector Laboratories, USA) and at least 300 spermatozoa per sample were scored by one experienced observer with an epifluorescence microscope (×40, Leica Microsystems, Wetzlar, Germany) [70]. The proportion of spermatozoa with a damaged DNA molecule is expressed as a percentage.

### 4.10. TBARS Assay

An aliquot of each sample was centrifuged (800× *g*, 10 min) and the sperm pellet was sonicated (28 kHz, 30 s) in the presence of the RIPA buffer (Sigma-Aldrich, St. Louis, MO, USA) and protease inhibitor (Sigma-Aldrich, St. Louis, MO, USA). Following the second round of centrifugation (11,828× *g*, 4 °C, 10 min) and purification, the lysates were subjected to the quantification of malondialdehyde (MDA), considered to be the principal marker of lipid peroxidation (LPO).

The sperm lysates were pre-treated with 5% sodium dodecyl sulphate (Sigma-Aldrich, St. Louis, MO, USA) and subsequently exposed to 0.53% thiobarbituric acid (TBA; Sigma-Aldrich, St. Louis, MO, USA) dissolved in 20% acetic acid (pH 3.5; Centralchem, Bratislava, Slovakia) under high-temperature conditions (90–100 °C) for 1 h. Afterwards, the samples were cooled down for 10 min and centrifuged (1750× *g*, 10 min). The supernatants (150 μL) were transferred into a transparent 96-well plate and the levels of MDA were assessed at 540 nm using the Glomax microplate spectrophotometer (Promega, Madison, WI, USA). MDA levels are expressed as μmol/L [71].

### 4.11. Statistical Analysis

All data were processed with the GraphPad Prism statistical program (version 9.0.0. for Mac; GraphPad Software, San Diego, CA, USA). First, all data were subjected to the Shapiro-Wilk normality test considering a normal (Gaussian) distribution. All data sets passed the normality test with non-significant results at the alpha level of 0.05. Descriptive statistical characteristics (mean, standard error of the mean), One-way ANOVA and Dunnett’s post-test were chosen for subsequent analyses. The experimental groups were compared with the control. The level of significance was set at **** *p* < 0.0001; *** *p* < 0.001; ** *p* < 0.01; * *p* < 0.05.

## Figures and Tables

**Table 1:** Motility (MOT) of human spermatozoa (*n* = 51) exposed to NTP of varying time intervals.

MOT [%]	0 s	15 s	30 s	60 s	90 s
0 h	72.08 ± 1.69	71.79 ± 2.23	71.11 ± 2.55	62.22 ± 1.99 *	49.22 ± 1.97 **
2 h	65.77 ± 2.63	63.99 ± 3.12	63.15 ± 3.51	50.81 ± 3.13 *	37.03 ± 4.05 ***

Mean ± SEM. * *p* < 0.05; ** *p* < 0.01; *** *p* < 0.001. Lines: times of NTP exposition. Columns: evaluation times.

**Table 2:** Progressive motility (PROG) of human spermatozoa (*n* = 51) exposed to NTP of varying time intervals.

PROG [%]	0 s	15 s	30 s	60 s	90 s
0 h	40.33 ± 2.20	40.38 ± 2.47	35.83 ± 2.65	24.88 ± 2.87 **	16.79 ± 2.74 ***
2 h	34.08 ± 3.02	30.38 ± 2.71	27.00 ± 2.39	20.25 ± 3.16 ***	13.42 ± 3.15 ***

Mean ± SEM. ** *p* < 0.01; *** *p* < 0.001. Lines: times of NTP exposition. Columns: evaluation times.

**Table 3:** Path velocity (VAP) of human spermatozoa (*n* = 51) exposed to NTP of varying time intervals.

VAP [µm/s]	0 s	15 s	30 s	60 s	90 s
0 h	54.32 ± 2.41	52.56 ± 0.20	51.56 ± 1.73	39.70 ± 1.68 ***	34.89 ± 1.98 ***
2 h	46.26 ± 3.83	42.74 ± 1.92	40.32 ± 2.35	36.13 ± 1.89 ***	30.79 ± 3.57 ***

Mean ± SEM. *** *p* < 0.001. Lines: times of NTP exposition. Columns: evaluation times.

**Table 4:** Curvilinear velocity (VCL) of human spermatozoa (*n* = 51) exposed to NTP of varying time intervals.

VCL [µm/s]	0 s	15 s	30 s	60 s	90 s
0 h	85.53 ± 3.06	80.28 ± 2.35	80.20 ± 2.24	62.98 ± 1.63 ****	57.83 ± 2.22 ****
2 h	72.76 ± 4.71	68.99 ± 2.51	67.47 ± 2.35	50.90 ± 1.69 ****	50.73 ± 4.11 ****

Mean ± SEM. **** *p* < 0.0001. Lines: times of NTP exposition. Columns: evaluation times.

**Table 5:** Straight line velocity (VSL) of human spermatozoa (*n* = 51) exposed to NTP of varying time intervals.

VSL [µm/s]	0 s	15 s	30 s	60 s	90 s
0 h	46.89 ± 2.53	44.16 ± 2.26	42.81 ± 1.89	32.29 ± 2.18 ***	27.26 ± 2.53 ****
2 h	38.78 ± 4.13	32.71 ± 2.25	31.62 ± 2.69	25.52 ± 2.31 ***	20.28 ± 4.04 ****

Mean ± SEM. *** *p* < 0.001; **** *p* < 0.0001. Lines: times of NTP exposition. Columns: evaluation times.

**Table 6:** Straightness (STR) of human spermatozoa (*n* = 51) exposed to NTP of varying time intervals.

STR [%]	0 s	15 s	30 s	60 s	90 s
0 h	83.00 ± 1.94	82.44 ± 1.52	81.56 ± 1.59	79.00 ± 2.25	75.89 ± 3.01 *
2 h	80.44 ± 2.68	78.58 ± 1.89	77.51 ± 2.05	76.32 ± 3.08	64.56 ± 6.83 *

Mean ± SEM. * *p* < 0.05. Lines: times of NTP exposition. Columns: evaluation times.

**Table 7:** Lateral amplitude (ALH) of human spermatozoa (*n* = 51) exposed to NTP of varying time intervals.

ALH [µm]	0 s	15 s	30 s	60 s	90 s
0 h	3.85 ± 0.18	3.85 ± 0.19	3.44 ± 0.29	3.44 ± 0.023	2.84 ± 0.24 *
2 h	3.57 ± 0.20	3.43 ± 0.14	3.43 ± 0.13	2.91 ± 0.41	2.76 ± 0.41 *

Mean ± SEM. * *p* < 0.05. Lines: times of NTP exposition. Columns: evaluation times.

**Table 8:** Beat cross frequency (BCF) of human spermatozoa (*n* = 51) exposed to NTP of varying time intervals.

BCF [Hz]	0 s	15 s	30 s	60 s	90 s
0 h	28.39 ± 1.09	26.81 ± 0.97	26.56 ± 1.22	24.53 ± 1.16 *	24.03 ± 0.82 *
2 h	25.50 ± 1.12	24.10 ± 2.31	23.93 ± 2.27	21.91 ± 1.22 *	21.13 ± 3.11 *

Mean ± SEM. * *p* < 0.05. Lines: times of NTP exposition. Columns: evaluation times.

**Table 9:** Linearity (LIN) of human spermatozoa (*n* = 51) exposed to NTP of varying time intervals.

LIN [%]	0 s	15 s	30 s	60 s	90 s
0 h	55.11 ± 2.29	54.89 ± 2.48	54.00 ± 2.81	51.00 ± 2.50	47.22 ± 3.32
2 h	52.56 ± 3.28	50.44 ± 2.11	49.78 ± 2.33	49.11 ± 2.93	45.44 ± 2.62

Mean ± SEM. Lines: times of NTP exposition. Columns: evaluation times.

**Table 10:** Apoptotic changes to the membrane of human spermatozoa (*n* = 51) exposed to NTP of varying time intervals.

AV-Positivity [%]	0 s	15 s	30 s	60 s	90 s
0 h	10.97 ± 0.99	11.07 ± 2.00	12.47 ± 1.99	15.77 ± 2.88	17.66 ± 2.57 *
2 h	13.27 ± 2.08	14.02 ± 1.45	17.08 ± 2.12	22.89 ± 3.05 *	28.30 ± 2.75 ***

AV—Annexin V. Mean ± SEM. * *p* < 0.05; *** *p* < 0.001. Lines: times of NTP exposition. Columns: evaluation times.

**Table 11:** Necrotic changes to the membrane of human spermatozoa (*n* = 51) exposed to NTP of varying time intervals.

PI-Positivity [%]	0 s	15 s	30 s	60 s	90 s
0 h	5.55 ± 0.99	6.12 ± 1.34	6.76 ± 1.96	10.15 ± 1.92 *	12.06 ± 1.99 **
2 h	7.99 ± 1.97	8.32 ± 2.05	9.07 ± 2.19	15.55 ± 2.14 *	19.22 ± 2.55 **

PI—Propidium iodide. Mean ± SEM. * *p* < 0.05; ** *p* < 0.01. Lines: times of NTP exposition. Columns: evaluation times.

**Table 12:** Mitochondrial activity of human spermatozoa (*n* = 51) exposed to NTP of varying time intervals.

MTT Test [%]	0 s	15 s	30 s	60 s	90 s
0 h	100.00 ± 0.00	109.01 ± 7.11	103.00 ± 8.33	102.20 ± 6.78	94.44 ± 7.79
2 h	100.00 ± 0.00	110.20 ± 8.87	104.42 ± 7.53	90.66 ± 9.17	88.62 ± 9.49

Mean ± SEM. Lines: times of NTP exposition. Columns: evaluation times.

**Table 13:** The mitochondrial membrane potential of human spermatozoa (*n* = 51) exposed to NTP of varying time intervals.

Δψm [Red/Green Ratio]	0 s	15 s	30 s	60 s	90 s
0 h	3.26 ± 0.30	3.23 ± 0.31	3.20 ± 0.34	3.00 ± 0.28	2.74 ± 0.19 *
2 h	2.39 ± 0.21	2.33 ± 0.17	2.22 ± 0.23	1.56 ± 0.17 *	1.25 ± 0.09 **

Mean ± SEM. * *p* < 0.05; ** *p* < 0.01. Lines: times of NTP exposition. Columns: evaluation times.

**Table 14:** DNA fragmentation of human spermatozoa (*n* = 51) exposed to NTP of varying time intervals.

DNA Fragmentation Index [%]	0 s	15 s	30 s	60 s	90 s
0 h	12.84 ± 2.60	13.94 ± 1.91	14.77 ± 1.92	26.55 ± 3.26 ***	27.62 ± 3.53 ***
2 h	18.98 ± 3.10	20.02 ± 2.10	22.21 ± 2.41	39.60 ± 3.12 ***	48.92 ± 3.90 ***

Mean ± SEM. *** *p* < 0.01. Lines: times of NTP exposition. Columns: evaluation times.

**Table 15:** Reactive oxygen species (ROS) production by human spermatozoa (*n* = 51) exposed to NTP of varying time intervals.

ROS [RLU/s/10^6^ sperm]	0 s	15 s	30 s	60 s	90 s
0 h	25.54 ± 3.18	27.93 ± 3.55	29.34 ± 4.00	37.33 ± 3.87 *	45.28 ± 4.62 **
2 h	29.99 ± 4.07	32.33 ± 4.22	34.41 ± 3.57	42.44 ± 4.71 *	51.88 ± 4.86 **

Mean ± SEM. * *p* < 0.05; ** *p* < 0.01. Lines: times of NTP exposition. Columns: evaluation times.

**Table 16:** Superoxide production by human spermatozoa (*n* = 51) exposed to NTP of varying time intervals.

NBT Test [%]	0 s	15 s	30 s	60 s	90 s
0 h	100.00 ± 0.00	102.44 ± 7.79	102.20 ± 6.78	106.08 ± 9.76	107.09 ± 9.03
2 h	100.00 ± 0.00	107.88 ± 7.77	109.22 ± 8.98	116.96 ± 9.92	124.03 ± 9.19

Mean ± SEM. Lines: times of NTP exposition. Columns: evaluation times.

**Table 17:** Lipid peroxidation of human spermatozoa (*n* = 51) exposed to NTP of varying time intervals.

MDA [μmol/L]	0 s	15 s	30 s	60 s	90 s
0 h	0.62 ± 0.08	0.63 ± 0.06	0.67 ± 0.07	0.77 ± 0.08	0.88 ± 0.09 *
2 h	0.77 ± 0.09	0.79 ± 0.07	0.84 ± 0.09	1.09 ± 0.12 *	1.32 ± 0.13 **

Mean ± SEM. * *p* < 0.05; ** *p* < 0.01. Lines: times of NTP exposition. Columns: evaluation times.

## Data Availability

The data presented in this study are available on request from the corresponding author.
